# Seroprevalence of anti-hepatitis E virus (HEV) in a Korean population: comparison of two commercial anti-HEV assays

**DOI:** 10.1186/1471-2334-12-142

**Published:** 2012-06-22

**Authors:** Hyun Kyung Park, Sook-Hyang Jeong, Jin-Wook Kim, Byung-Hyun Woo, Dong Ho Lee, Hyun Young Kim, Soyeon Ahn

**Affiliations:** 1 Department of Internal Medicine, Gumi-dong 300, Bundang-gu, Seongnam-si, Gyeonggi-do, South Korea; 2 Health Promotion Center, Gumi-dong 300, Bundang-gu, Seongnam-si, Gyeonggi-do, South Korea; 3 Medical Research Collaborating Center, Seoul National University Bundang Hospital, Gumi-dong 300, Bundang-gu, Seongnam-si, Gyeonggi-do, South Korea; 4 Department of Internal Medicine, Seoul National University Bundang Hospital, Gumi-dong 300, Bundang-gu, Seongnam-si, Gyeonggi-do, South Korea

**Keywords:** Hepatitis E virus, Seroprevalence, Anti-HEV assay, Korea

## Abstract

**Background:**

Hepatitis E virus (HEV) has emerged as an important cause of epidemic and sporadic acute viral hepatitis worldwide. This study investigated the seroprevalence of anti-HEV in a Korean population and compared the performance of two commercially available anti-HEV assays.

**Methods:**

A total 147 health-check examinees were randomly sampled as matched to the age- and sex- adjusted standard population based on the Korean National Census of 2007. Serum immunoglobulin G anti-HEV was determined by using the Genelabs assay (Genelabs, Singapore) and the Wantai assay (Wantai, Beijing, China).

**Results:**

The overall anti-HEV seroprevalence was 23.1% (95% CI, 16.1-30.1%) using the Wantai assay and 14.3% (95% CI, 8.3-20.3%) using the Genelabs assay. Only 12 samples (8.1%) were positive for anti-HEV as measured by both assays; agreement between the two assays was poor (kappa value of 0.315). The anti-HEV seroprevalence increased with age from 2% and 3% in the people younger than 20-years-of-age to 34.6% and 42.3% in those over 59-years-of-age by the Genelabs and Wantai assay, respectively.

**Conclusions:**

The HEV seroprevalence in Korean population is about 20% overall, with seroprevalence increasing in this population with increasing age. There was poor concordance in the results of the Genelabs and Wantai assays, which warrants further study concerning a reliable diagnostic test for the diagnosis of hepatitis E.

## Background

Hepatitis E virus (HEV) is an emerging disease of global importance as a major cause of enterically transmitted hepatitis [1]. After its discovery in 1983 [2], HEV was characterized as a non-enveloped, single stranded, positive sense RNA virus classified as a member of the Hepeviridae family, Hepevirus genus.

The epidemiology of HEV infection displays two patterns. The first is an outbreak pattern in areas of high endemicity (primarily via water-borne or fecal-to-oral transmission). The second is a sporadic pattern that occurs worldwide via zoonotic transmission and food borne transmission. Hepatitis E is a serious public health problem responsible for over 50% of acute viral hepatitis cases [3] in endemic countries, which includes large parts of Asia, Africa, the Mediterranean region, Mexico, and South America [4]. In contrast, HEV infection has previously been considered rare in developed countries [5], but is far more common than previously recognized [6,7]. Zoonotic transmission, especially from pigs, has been suggested [8]. However, the true burden of infection and its implication on public health impact remain undefined.

In developed countries, anti-HEV immunoglobulin G (IgG) prevalence rates range between 3% to above 20% [7,9,10]. These appear to be higher than those expected from the low rate of clinically evident hepatitis E disease in developed countries, suggesting that subclinical or unrecognized infection is common [11]. Although several cases of imported and locally acquired hepatitis E have been reported in Korea, the anti-HEV seroprevalence data have not been available due to lack of disease recognition and to the limited availability of diagnostic tools [12].

Several anti-HEV assays have been developed and are available for use. However, the performance of each anti-HEV assay has not been well studied. One study [13] reported highly variable results among the different assays, which suggested that the diagnosis of HEV infection using anti-HEV tests should be made with caution. Findings from a comparison of two commercially available IgG anti-HEV enzyme-linked immunosorbent assay (EIA) kits – the Genelabs EIA (Genelabs, Singapore) and the Wantai EIA (Wantai Biological Pharmacy Enterprise, Beijing, China) – demonstrated that the Wantai EIA was more sensitive than the Genelabs EIA, and produced positive results for a longer time post-infection [14]. Despite these findings, the Genelabs anti-HEV EIA remains the more popular assay.

The aims of this study were to investigate the seroprevalence of anti-HEV and its related factors in a Korean population, and to again compare the results of the two aforementioned commercially available serological assays for the detection of HEV-specific IgG.

## Methods

### Subjects and serum samples

A total of 484 health-check examinees visiting the Health Promotion Center of Seoul National University Bundang Hospital from June 2006 to September 2006 and agreed to participate in this study were enrolled. Among them, 147 sera were randomly selected by matching the subjects to the age- and sex- adjusted standard population of the Korean National Census of 2007. In detail, 484 subjects were first allocated into the each category of age in decades and sex, and then 147 were randomly selected according to the proportions of the standard population in each category. Serum samples were stored at −70°C until the analysis. Informed consent was obtained from all participants and the study protocol was approved by the Institutional Review Board of Seoul National University Bundang Hospital.

### Measurement of anti-HEV IgG using the two commercial assays

The Genelabs HEV IgG enzyme-linked immunosorbent assay (ELISA) and the Wantai HEV IgG ELISA were used to detect IgG anti-HEV in sera. The Genelabs assay detects antibodies directed at a mixture of recombinant peptides specified by the open reading frame (ORF) 2 and ORF 3 obtained from two different strains of HEV: one from Mexico (genotype 2) and the other from Burma (genotype 1) [15]. The Wantai assay uses a recombinant peptide corresponding to amino acid residues 396–606 of the major structural protein specified by ORF2 derived from a Chinese isolate of HEV (genotype 4) [16]. Anti-HEV IgG in all serum samples were examined with both the Genelabs and Wantai assay, according to manufacturer’s instructions, with three negative and two positive control wells included on each plate. The optical density (OD) of each sample was determined at 450 nm. Samples with an OD greater than the cut-off value were determined to be positive. All serum samples were tested in duplicate and the cut-off value was calculated to be the mean absorbance value of the negative control plus 0.5 for the Genelabs assay and plus 0.16 for the Wantai assay, according to the manufacturer’s instructions.

### Statistical analyses

Descriptive statistics were reported and agreement between two assays was quantified with the kappa statistic. The chi-squared test and paired *T*-test were used to determine whether any patient characteristics were associated with different results in the two assays. Differences were considered to be statistically significant at *P* < 0.05. The statistical analyses were carried out using the SPSS statistical program, version (SPSS,Chicago, IL, USA).

## Results

The study subjects included 72 males and 75 females, with an overall mean age of 45 years. These characteristics were compatible with the data from the Korean National Census of 2007. The mean body mass index of the subjects was 23.0, and the mean serum levels of alanine aminotransferase and total bilirubin were 28.4 IU/mL and 1.0 mg/dL, respectively. The hepatitis B virus surface antigen (HBsAg) positive rate was 3.8%, and the anti-hepatitis C virus antibody positive rate was zero.

The overall seroprevalence of anti-HEV IgG in the population, which was age- and sex-adjusted to the standard population of Korea according to 2007 Census results, was 23.1% (34/147; 95% CI, 16.1-30.1%) using the Wantai assay and 14.3% (21/147; 95% CI, 8.3-20.3%) using the Genelabs assay. The comparison of the seropositive samples of anti-HEV measured using the two assays are summarized in Table 1. Twelve subjects (8.2%) were positive and 104 subjects were negative for anti-HEV measured by both assays. The agreement between the Wantai and the Genelabs assay was poor, with a κ value of 0.315.

**Table 1:** Comparison of seropositivity of anti-HEV IgG measured using the two immunoassays

**Anti-HEV assays**	**Wantai positive**	**Wantai negative**	**Total, n (%)**
	**n (%)**	**n (%)**	
Genelabs positive	12 (8.2)	9 (6.1)	21 (14.3)
n (%)			
Genelabs negative	22 (15.0)	104 (70.7)	126 (85.7)
n (%)			
Total, n (%)	34 (23.1)	113 (77.9)	147 (100)

*n*, number.

A κ value of 0.315 for the agreement between the Wantai and the Genelabs assay results.

**Figure 1:**
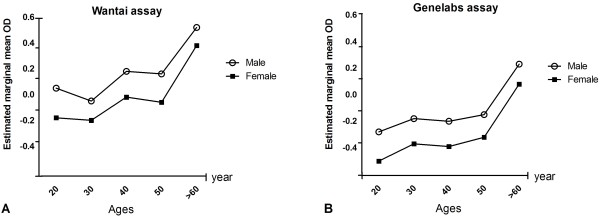
**The estimated mean values of optical density (OD) of anti-HEV IgG measured using Wantai and Genelabs immunoassays according to age and gender in a Korean adult population.** A, B. The mean OD values of anti-HEV IgG showed significant increase according to the increase of the age in both assays (*P* value = 0.002 in the Wantai assay, *P* value = 0.003 in the Genelabs assay; *P* values calculated by ANOVA). The overall OD values were higher in men than woman, with no significant difference between the two in both assays.

Data on the anti-HEV seropositive rates according to age groups and gender are summarized in Table 2. The anti-HEV positive rates increased significantly according to the increase of the age (*P* = 0.011 in the Wantai assay, *P* = 0.029 in the Genelabs assay). The anti-HEV seropositive rate in males tended to be higher than that in females for both assays (*P* = 0.089 in the Wantai assay, *P* = 0.201 in the Genelabs assay). The mean OD values also showed a higher tendency in older age group and men in both assays (Figure 1). There was no relationship between the anti-HEV seropositivity and the presence of hepatitis B or C markers, or ALT level.

**Table 2:** Anti-HEV seropositive rates according to age groups and gender

	**Number tested**	**Seropositivities for :**			
		**Wantai % (95% CI)**	**P-value**	**Genelabs % (95% CI)**	**P-value**
Age group, years			0.011		0.029
20-29	24	12.5 (0–25.5)		8.3 (0–19.3)	
30-39	34	11.8 (0.8-22.8)		11.8 (0.8-22.8)	
40-49	41	19.5 (0–31.5)		9.8 (0.8-18.8)	
50-59	22	31.8 (12.8-50.8)		9.1 (0–21.1)	
> 59	26	46.1 (27.1-65.1)		34.6 (16.6-52.6)	
Gender			0.089		0.201
male	72	29.1 (19.1-39.1)		18.1 (9.1-27.1)	
female	75	17.3 (9.3-25.3)		11.7 (4.7-18.7)	
Total	147	23.1 (16.1-30.1)		14.3 (8.3-20.3)	

*CI*, confidence interval.

*P* values by chi-square test.

## Discussion

In this study, we evaluated the anti-HEV IgG positive rate in a Korean adult population composed of 147 health check examinees, age- and sex-adjusted to the standard population based on the Korean National Census of 2007. The seropositivity of anti-HEV in our study subjects was 23.1% in the Wantai assay and 14.3% in the Genelabs assay. The agreement of the results between assays was poor, with a κ value of 0.315. The anti-HEV positivity and mean OD values of anti-HEV measured using both assays significantly increased with increasing age.

Korea is not an endemic area of hepatitis E, and less than 20 cases of acute hepatitis E cases have been reported in South Korea since 2002 [17-20]. Most of these cases were of Korean origin rather than having been imported from highly endemic areas. Among them, only a few cases demonstrated HEV RNA, which identified HEV genotype 3 and genotype 4, and genotype 3 HEV sequences isolated from human cases were nearly identical to those from pigs in Korea. Moreover, we recently reported a case of genotype 4 HEV hepatitis after ingestion of raw bile juice of wild boar, suggesting zoonotic transmission of HEV in Korea [21,22]. Therefore, at least two HEV genotypes already circulate in Korea, and it is likely that more cases will be identified with the increased recognition of HEV.

Previous studies on the seropositivity of anti-HEV in Korea reported a positive rate between 8% and 17% in the various populations, based on blood donors, or healthy adults visiting some diagnostic laboratories. However, those study subjects had not been adjusted to the standard population and even no detailed demographic information had been provided; all these preliminary studies had only used the Genelabs assay [23-25]. Therefore, direct comparison of our data to the previous results was difficult. Recently, the comparative seroprevalence in 1,500 people over 40-years-of-age living in Japan, Korea, and China were reported using anEIA developed in Japan [26]. The anti-HEV positivity was 50.7% in Korean Chinese, 34% in South Koreans, and 14.3% in Koreans living in Japan. In our study, the seroprevalence of anti-HEV in adults over 40-years-of-age was 16.9% in the Genelabs assay and 30.3% in the Wantai assay, which was similar to the previous results.

Presently, older age groups tended to have higher HEV seroprevalence rates by both assays, and the differences were statistically significant. Age has been correlated with higher HEV seroprevalence rates [27]. IgG anti-HEV titers remain high from 1–4.5 years after the acute phase [28], and one study detected anti-HEV IgG in about 47% of individuals 14 years after acute HEV infection [29]. These persistent anti-HEV IgG can account for the high rates of seroprevalence in older subjects among the general population [30]. Therefore, differences in seroprevalence rates between different populations must be interpreted with caution [31], because demographic variables, such as age, are related to the prevalence, and because the assays vary in their sensitivity [1] in the absence of standardized commercially available confirmatory assays, such as reverse transcription-PCR.

Several commercial serological assays for the detection of anti-HEV IgG are available [32]. Among them, the Genelabs EIA has been the most commonly used worldwide. Its antigens use polypeptides from the C-terminal ORF3 and ORF2 domains of HEV genotypes 1 and 2 [15]. The Genelabs assay, based on genotypes 1 and 2, vary greatly in sensitivity (50%-90%) despite an excellent specificity (93%-100%) [15,33-35]. Moreover, the experience and test performance with this assay has come mainly from regions of high endemicity and western countries. In our results, the agreement between the Wantai and the Genelabs assays was poor (κ value = 0.315), and the Wantai assay displayed higher seropositivity than the Genelabs assay, which may suggest the higher sensitivity of the Wantai assay. However, we did not use the standard serum in our study, so that the sensitivity and specificity in our study cannot be assessed. Recently, Bendall et al. [14] compared the performance of Genelabs and Wantai HEV IgG EIA kit using World Health Organization standard sera; the Wantai assay was more sensitive than the Genelabs assay, and continued to test infected individuals as positive for longer periods post-infection. The authors also tested 500 blood samples obtained from blood donors in the United Kingdom using both assays; the Wantai assay resulted in a substantially higher estimate of seroprevalence (16.2%) than that of Genelabs (3.6%) [14]. Moreover, the Wantai kit has been reported to be more sensitive because the peptides used in the Wantai assay may associate into dimers, which react more strongly with HEV reactive sera than the linear monomeric antigens used in the Genelabs assay [36]. The Wantai anti-HEV IgG ELISA uses a peptide encoded by a structural region of ORF-2 of HEV genotype 4. Specificity is difficult to assess in situations other than acute hepatitis E because there is no gold standard for checking the specificity of the current anti-HEV ELISA. Both assays have been compared to Western blots and other assays in population-based studies. The specificity of the Genelabs assay was 97% [37] and that of the Wantai assay was 99.6% [16,38]. Accepting that both assays have similar specificities, it is reasonable to assume that the Wantai assay gives a more reliable estimate of anti-HEV seropositivity rates than the Genelabs assay.

The results of this study should be interpreted in the context of its limitations. First, the data were obtained from a single center using cross-sectional design, so that time trends could not be addressed. However, direct measurement of incidence of hepatitis E is difficult because infection is most often asymptomatic and unrecognized. Second, we were unable to assess the distribution of risk factors in our study population, such as profession, hobbies, diet, social status, residence, or travel history. Further studies are required to clarify the epidemiology and risk factors for HEV infection in Korea.

## Conclusions

In conclusion, the prevalence of anti-HEV in Korean adult population was about 20%, with higher prevalence at increased age. However, different assays for the detection of anti-HEV IgG result in significantly different results. Therefore, future studies on the development of standard diagnostic tests and their validation are warranted.

## Competing interests

The authors declare that they have no competing interests.

## Authors’ contributions

SHJ designed the study and helped to draft the manuscript. HKP and BHW carried out the immunoassays. DLH and HYK collected clinical data. HKP drafted the manuscript. JWK participated in its design and coordination. SYA performed the statistical analysis. All authors read and approved the final version of this manuscript.

## Pre-publication history

The pre-publication history for this paper can be accessed here:

http://www.biomedcentral.com/1471-2334/12/142/prepub
